# Diagnosis of post-myocardial infarction left ventricular rupture on CT

**DOI:** 10.1259/bjrcr.20220008

**Published:** 2022-04-25

**Authors:** Kimberley Yu San Lee, Pragya Attri, Peace I Tamuno, Ee Lyn Au, Barbara E Hochstein

**Affiliations:** 1 Department of General Medicine, Rotorua Hospital, Rotorua, New Zealand; 2 Department of General Medicine and Cardiology, Rotorua Hospital, Rotorua, New Zealand; 3 Department of Emergency Medicine, Rotorua Hospital, Rotorua, New Zealand; 4 Department of Radiology, Rotorua Hospital, Rotorua, New Zealand

## Abstract

Myocardial rupture is often a catastrophic complication of acute myocardial infarction. Diagnosis can be challenging in the critically unwell patient. We present the case of a 70-year-old female who collapsed in the community with pulseless electrical activity, in cardiac arrest. She was transferred emergently to hospital where early resuscitation efforts were suggestive of a posterior myocardial infarct and severe blood loss. Point-of-care cardiac ultrasound demonstrated pericardial effusion but could not rule out aortic dissection. The patient underwent CT imaging with intravenous contrast which revealed left ventricular rupture secondary to the infarction. CT imaging can be a valuable diagnostic adjunct in patients with suspected post-infarction myocardial rupture.

## Introduction

Myocardial rupture remains one of the most devastating sequelae of acute myocardial infarction (MI). Risk factors include female sex, older age, hypertension, first MI, and not receiving percutaneous coronary intervention (PCI).^1–3^ Recent observational data suggest that cardiac rupture is more likely to occur within 24 to 48 h of an acute MI.^2,3^ Even with prompt recognition, myocardial rupture can rapidly lead to electromechanical dissociation, cardiac tamponade, and sudden death.^1,2^ We describe the clinical course and diagnosis of a patient with cardiac arrest secondary to myocardial rupture. Although the patient could not be successfully resuscitated, the CT imaging obtained provided confirmation and closure as to the cause of her unexpected death.

## Clinical presentation

A 70-year-old female was transferred to the Emergency Department (ED) of a non-PCI capable hospital by ambulance, having just achieved return of spontaneous circulation (ROSC) after community cardiac arrest. The attending paramedics reported that she had been out walking her dog that morning and had collapsed. Bystanders gave 10 min of cardiopulmonary resuscitation (CPR) before the paramedics arrived and confirmed the patient was in pulseless electrical activity (PEA) arrest. ROSC was achieved after 20 min with the patient in PEA throughout. The patient was intubated during the community resuscitation.

Collateral history from existing medical notes and the family revealed that the patient had a background of treated essential hypertension and knee osteoarthritis, for which she had been engaging in community exercises. She had mentioned a minor heartburn sensation the night before but had otherwise been well.

On arrival to ED, the patient deteriorated and became pulseless with blood pressure (BP) of 61/10. CPR was recommenced for 4 min with successful ROSC.

**Figure 1.**
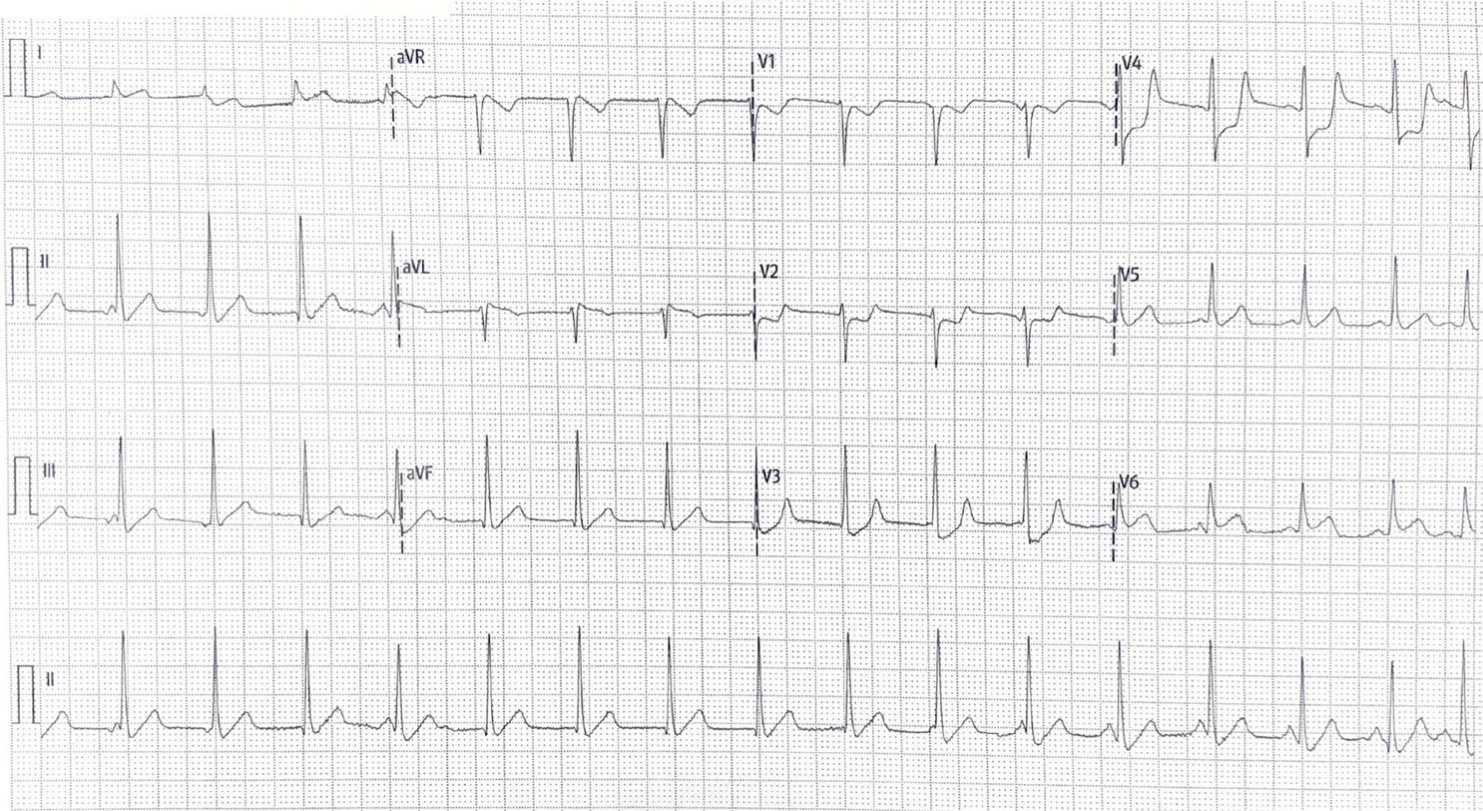
ECG showing ST segment depression in leads V1-V4, concerning for inferobasal MI. There is also ST segment elevation in lead V6, which would suggest right coronary artery (RCA) MI where the RCA is the dominant vessel. Due to the patient’s haemodynamic instability, a posterior ECG could not be obtained.

A 12-lead electrocardiogram (ECG) was obtained (Figure 1), which looked highly suspicious for a posterior STEMI.

The patient then became bradycardic with a heart rate of 30 bpm and blood pressure was lost again. CPR was recommended for around 20 min and ROSC was reattained. The patient was started on a noradrenaline and adrenaline infusion. Intravenous (IV) access had been very challenging thus far and preparations were being made for central access.

The patient was discussed with the nearest primary PCI-capable centre. It was agreed that given the extensive CPR and haemodynamic instability, she was not a candidate for thrombolysis. She was accepted for transfer with a view to PCI.

Central and arterial access was achieved by this point. Blood results revealed a haemoglobin of 60 g l^−1^. Blood was urgently requested and transfused. High sensitivity cardiac troponin I assay level was 2116 ng l^−1^. A point-of-care bedside echocardiogram revealed a pericardial effusion and left haemothorax. The differential of aortic dissection or rupture was high. The patient proceeded to CT imaging to guide the next steps in management.

## Investigations/Imaging Findings

CT was performed on a 64 slice GE Revolution scanner. The CT imaging was obtained as follows:

Scan 1. Helical low-dose non-contrast CT imaging from the aortic arch to diaphragm.

Scan 2. CT Aortic Angiogram (CTA) from hyoid bone to femoral artery bifurcations. Thin sliced 0.625 with slice interval of 0.5 mm. Intravenous contrast was administered through a 20G cannula in the left antecubital fossa, using a Transflux (multipatient) setup with a Medrad power injector. 70 ml of Omnipaque 350 (non-ionic contrast medium) was given at 3.5 ml s^−1^, followed by a saline flush of 40 ml at 3.5 ml s^−1^. The contrast dose was calculated at 1 mL/kg based on an approximate weight of 70 kg. The scan was manually triggered due to poor cardiac output.

Scan 3. Delayed CT angiogram. As a result of the patient’s low cardiac output, the initial scan had outrun the contrast. Therefore, the delayed imaging was performed at 3 min post-contrast injection.

**Figure 2.**
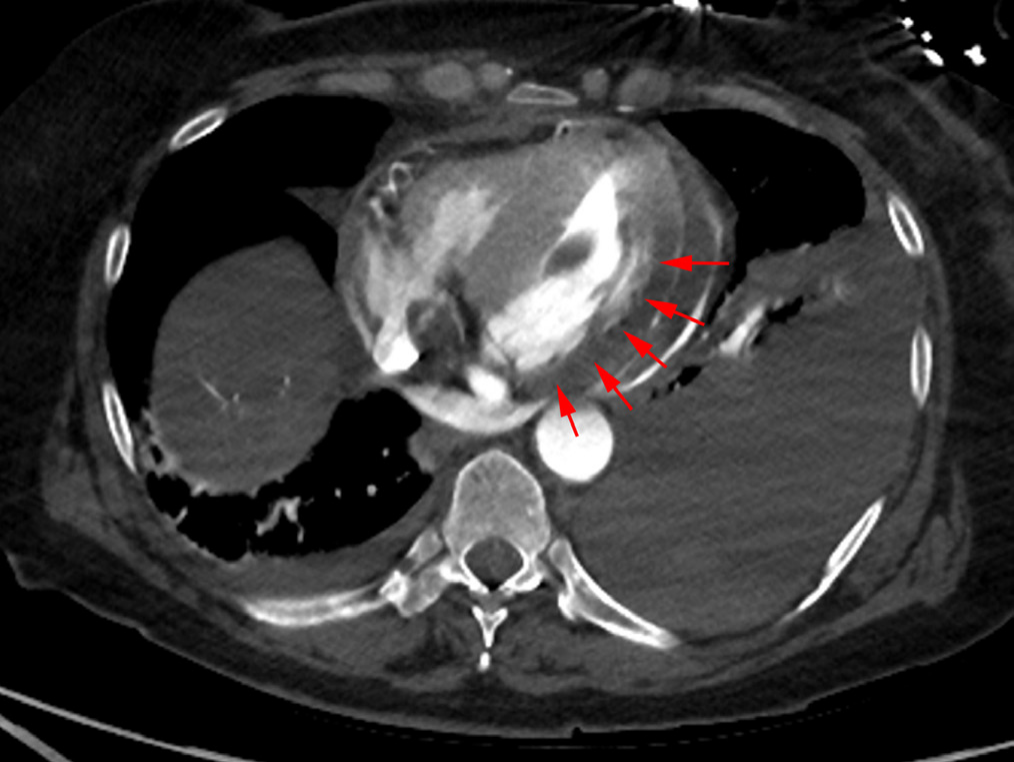
Axial image displaying decreased density of the posterolateral left ventricular wall (red arrows).

**Figure 3.**
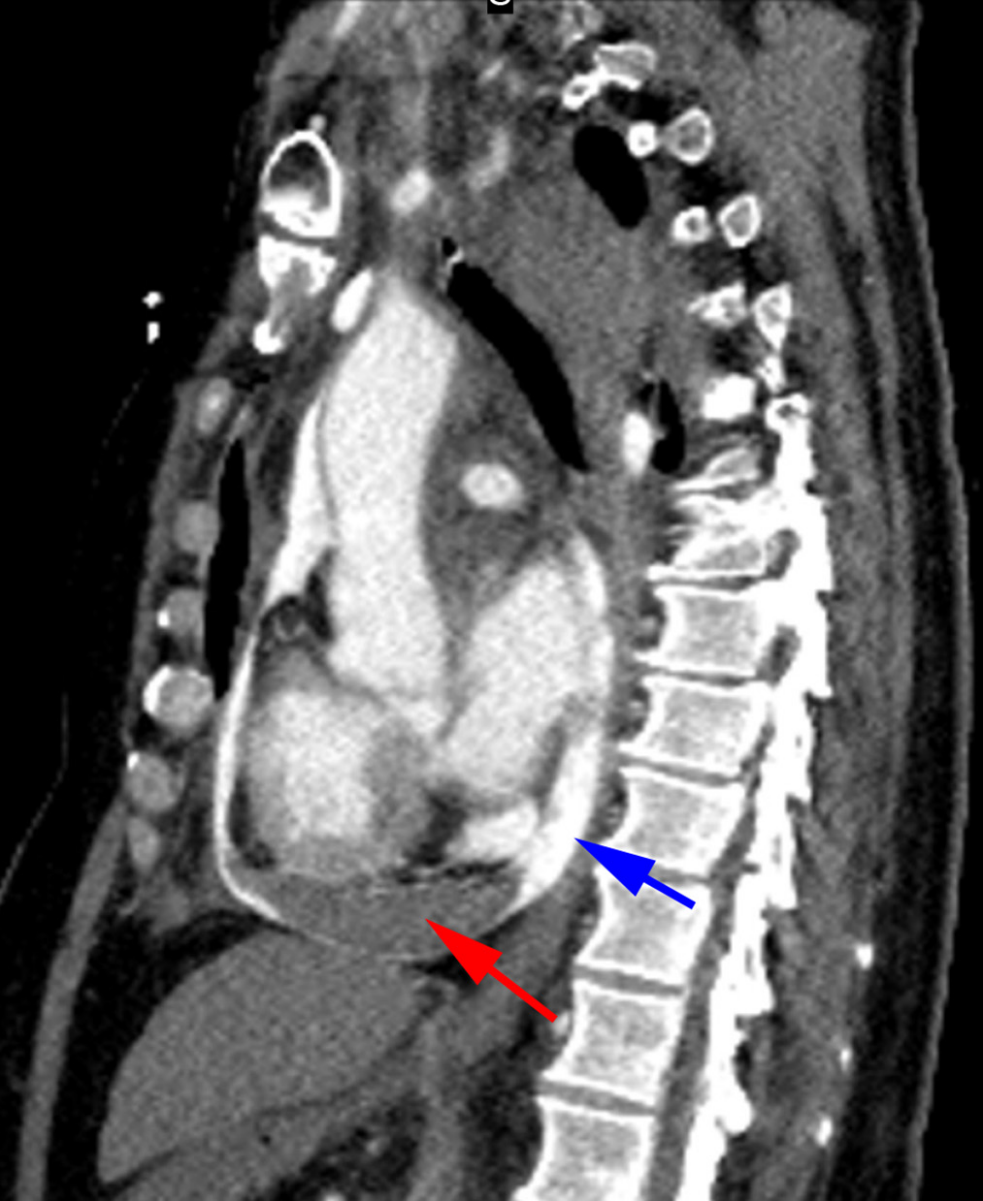
Sagittal view of contrast extravasating into the pericardial sac (blue arrow), consistent with arterial bleed from left ventricular rupture. The dense lens-shaped opacity located within the inferior pericardial sac measures 16 mm in width and indicates blood clot (red arrow).

The CT images demonstrate a myocardial infarct involving the posterolateral left ventricle (Figure 2). Extensive blood clot is seen within the pericardial space. Extravasation of contrast, indicating active arterial bleeding, is also captured flowing into the pericardial space (Figure 3). These findings are consistent with myocardial infarct and associated myocardial rupture. The left aortic arch is normal in contour with no evidence of aortic dissection.

**Figure 4.**
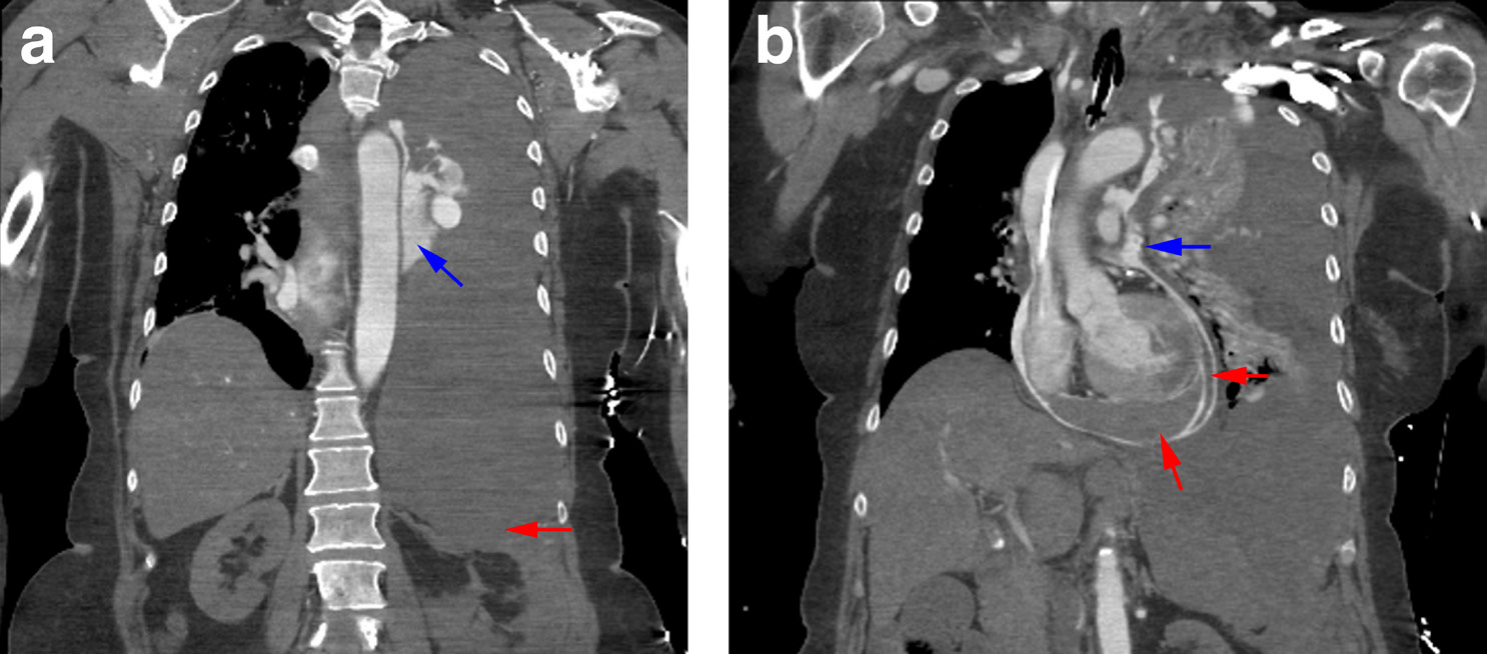
(**a**).Delayed coronal image showing contrast extravasation into the left hemithorax consistent with active arterial bleeding (blue arrow). The left hemidiaphgram is depressed and inverted due to the large haemothorax (red arrow). b. Delayed coronal frame demonstrating arterial blood rupture through the pericardium into the left hemithorax at the level of the main pulmonary artery (blue arrow). Two blood clots within the pericardial sac are again evident (red arrows).

Delayed imaging shows blood rupturing through the pericardium into the left pleural cavity at the level of the main pulmonary artery. There is an extensive left haemothorax. Marked gravity-dependent depression of the left hemidiaphragm indicates significant mass effect (Figure 4a and b).

## Treatment and outcome

The CT findings confirmed that the patient’s condition was not survivable. The family was informed, and the patient’s management was changed to supportive cares, on the end of life care pathway. She died later that day in the presence of her family.

## Discussion

Definitive management of myocardial rupture is generally only by surgical repair. Temporising measures to restore haemodynamic stability en route to emergency theatre include inotropic support, intravenous fluids and pericardiocentesis.^2,4^ Even with the availability of emergency surgery, mortality rates approach 90%.^1^

This patient was managed at a non-urban secondary care hospital without on-site access to Interventional Cardiology, Cardiothoracic or Vascular Surgery services. To receive specialist support, the patient would have had to be transferred to the nearest tertiary centre, a minimum of 60 min by helicopter. Her ongoing haemodynamic instability necessitated a more conclusive diagnosis to determine whether this would be possible.

Transthoracic echocardiogram is considered the ideal first-line diagnostic tool for suspected myocardial rupture.^5,6^ In the critical care setting, the echocardiogram is advantageous as a rapid investigation that can be performed at the bedside. However, the most common finding on echocardiogram is pericardial effusion, a non-specific sign which can also manifest as a result of acute aortic pathology.^5–7^ In cases of diagnostic uncertainty, CT with contrast can be a useful adjunct to definitively rule out aortic dissection and rupture.

On review of autopsy reports of patients who die after acute MI, prevalence of myocardial rupture is reported to be 30.7%,^8^ a significantly higher figure than the 1.8% reported in observational studies of the living.^1^ This patient underwent CT imaging that demonstrated a fatal condition. Although she could not be resuscitated, the images are highly valuable as they provided immediate answers to her treating physicians and her family. The contrast CT provided the closure needed to change the goals of care in a controlled manner from active intervention to comfort, whilst avoiding the need to consider an autopsy.

Myocardial rupture represents an ongoing challenge for all clinicians involved in the care of patients after MI. Due to the extreme acuity of most presentations of myocardial rupture, the diagnosis can be easily overlooked. Clinicians should be aware of risk factors and maintain high suspicion for rupture in patients with cardiovascular collapse and pericardial effusion. If used appropriately, contrast CT can provide diagnostic certainty and help guide treatment decisions.

## Learning points

- Myocardial rupture remains an uncommon but often fatal complication of acute MI, even in the modern PCI era.
- Clinicians should maintain a high index of suspicion for myocardial rupture in the setting of cardiovascular collapse with pericardial effusion.
- CT imaging in the appropriate clinical context can provide diagnostic certainty, guide ongoing management and avoid investigation by autopsy.
